# Stroma Insights: Potential Mechanism for Arsenic-Induced Prostate Cancer

**DOI:** 10.1289/ehp.124-A130

**Published:** 2016-07-01

**Authors:** Julia R. Barrett

**Affiliations:** Julia R. Barrett, MS, ELS, a Madison, WI–based science writer and editor, is a member of the National Association of Science Writers and the Board of Editors in the Life Sciences.

There is epidemiological evidence that exposure to inorganic arsenic may cause prostate cancer, but the molecular mechanisms to explain the potential relationship remain unclear.^1^ ^,^ ^2^ A new study in *EHP* looks beyond epithelial prostate cells—the cells most often studied in prostate cancer—to focus on stromal cells, a cell type that normally provides a supportive function. The findings suggest that inorganic arsenic primes these stromal cells to enhance the progression of cancer.^1^

Arsenic is naturally present in underground water, with hot spots of contamination worldwide.^3^ Drinking water from these aquifers is a primary source of arsenic exposure, and more than 200 million people are estimated to drink water that exceeds the World Health Organization’s recommended limit of 10 ppb arsenic.^3^ Chronic exposure has been associated with health problems ranging from skin lesions to various types of cancer, including prostate cancer.^2^ ^,^ ^3^ ^,^ ^4^ Both genetic and oxidative damage within the prostate have been suggested as possible mechanisms behind arsenic’s link with prostate cancer.^4^ ^,^ ^5^ According to the current study, the microenvironment surrounding the tumor may also have a role.^1^

**Figure:**
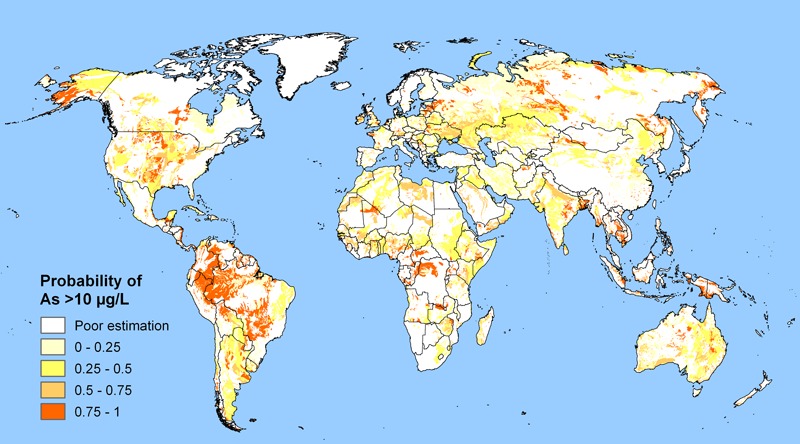
Worldwide, more than 200 million people are thought to drink water containing excessive amounts of arsenic. Based on: Amini et al. 2008b, Environ Sci Technol 42, 3669–3675

Like other glands, the prostate is surrounded by connective tissue, or stroma, containing several cell types that help maintain the gland’s normal growth and function.^6^ In the event of injury or infection, some stromal cells secrete molecules such as cytokines and growth factors to reduce inflammation, guard against infection, and foster healing.^7^ ^,^ ^8^ Tumor cells can provoke a similar reaction, but instead of restoring normalcy, the response may actually help the tumor cells thrive.^8^ ^,^ ^9^ ^,^ ^10^

Adipose-derived mesenchymal/stromal stem cells (ASCs) are particularly noteworthy in this context because they are drawn to tumors and concentrate in their vicinity.^9^ Given the concentration of ASCs in a tumor’s microenvironment, disruption of their normal function could affect tumor progression. Based on the evidence of a link between arsenic and prostate cancer,^4^ the authors hypothesized that arsenic could cause such disruption.

To test their hypothesis, the researchers conducted *in vitro* experiments with ASCs and a human prostate cancer cell line (PC3 cells). ASCs were cultured in media with 1, 10, or 75 ppb arsenic, and the media were analyzed to identify cytokines secreted by the cells. This analysis showed both up-regulation of tumor-promoting cytokines and down-regulation of tumor-suppressing cytokines, compared with unexposed cells. Proteomic analysis of the ASCs identified proteins whose expression was altered in the presence of arsenic, and further analysis uncovered a statistically significant relationship between increased expression of the protein heme oxygenase-1 and decreased expression of the protein thrombospondin-1. Both proteins are associated with the transforming growth factor-β signaling pathway,^8^ which plays a significant regulatory role in the progression of prostate cancer.^10^ A separate experiment confirmed that arsenic down-regulated this pathway in the ASCs.^1^

In other experiments, ASCs were cultured in media with 0 or 75 ppb arsenic, then washed and exchanged with arsenic-free media. This allowed for the collection of factors secreted by the ASCs. These conditioned media, minus the ASCs, were then used to culture PC3 cells. PC3 cell viability increased in media from arsenic-exposed but not nonexposed ASCs. The data collectively implied that arsenic causes ASCs to alter their response to tumor cells in a manner that enhances the growth of tumors.^1^

The researchers stress, however, that the study does not indicate that the stroma itself causes cancer. “It’s instead creating an environment that is more suitable for cancer to progress,” says lead author Joseph Shearer, a graduate student in pharmacology and toxicology at the University of Texas Medical Branch at Galveston.

Coauthor Marxa Figueiredo, an assistant professor of basic medical sciences at Purdue University, adds that cancer could be started by any number of factors, including genetic mutations or exposures to various other environmental contaminants. “In real life, we’re not impacted just by arsenic—we’re impacted by multiple mixtures of contaminants throughout our lives,” she says.

Although conducted in prostate cell culture, the study highlights a potentially important role of the stroma in other cancers. “We don’t have a complete understanding of how similar or different these stromal cells and microenvironments are between the different cancers,” says David Rowley, a professor of molecular and cellular biology at Baylor College of Medicine who was not involved in the study. “One could well imagine that exposure to something like arsenic would affect stroma in much the same way in many tissues. It wouldn’t surprise me if similar effects were seen in other tumor cell types.”

In fact, the impact of a dysregulated stroma could persist even in the absence of a tumor, according to Shearer. “When a tumor is excised, the reactive stroma could potentially still be relevant,” he says. “You have to think about not just treating the tumor, but also whether the environment is still aberrantly regulated.” With that in mind, the new findings highlight a possible route for improving cancer treatment someday.
